# Effects of the COVID-19 Lockdown on Body Composition and Bioelectrical Phase Angle in Serie A Soccer Players: A Comparison of Two Consecutive Seasons

**DOI:** 10.3390/biology10111175

**Published:** 2021-11-13

**Authors:** Francesco Campa, Tindaro Bongiovanni, Athos Trecroci, Alessio Rossi, Gianpiero Greco, Giulio Pasta, Giuseppe Coratella

**Affiliations:** 1 Department for Life Quality Studies, University of Bologna, 47921 Rimini, Italy; francesco.campa3@unibo.it; 2 Department of Biomedical Sciences for Health, University of Study of Milano, 20133 Milano, Italy; tindaro.bongiovanni@unimi.it (T.B.); athos.trecroci@unimi.it (A.T.); giuseppe.coratella@unimi.it (G.C.); 3 Department of Health, Nutrition and Exercise Physiology, Parma Calcio 1913, 43100 Parma, Italy; 4 Department of Computer Science, University of Pisa, 56126 Pisa, Italy; alessio.rossi@di.unipi.it; 5 Department of Basic Medical Sciences, Neuroscience and Sense Organs, University of Study of Bari, 70121 Bari, Italy; 6 Medical Department, Parma Calcio 1913, 43100 Parma, Italy; ghitopasta@hotmail.com

**Keywords:** BIA, BIVA, coronavirus disease, detraining, fat mass, football, muscle mass, team sports

## Abstract

**Simple Summary:**

In 2020, the first Italian soccer league (Serie A) was canceled due to the COVID-19 pandemic. Consequently, a detraining process was triggered in soccer players, leading coaches and sports scientists to implement alternative training strategies to prevent a remodeling in body composition. This study tested the hypothesis that male elite soccer players, when confined to their home during the coronavirus disease 2019 pandemic, will display unfavorable trends in bioelectrical and body composition parameters. The results of the present study showed that reduction in phase angle and muscle mass occurred in soccer players during the coronavirus disease 2019 pandemic lockdown. Recognizing these adverse effects of a detraining period is critical in avoiding adverse effects on body composition in soccer players. In addition, the bioelectrical phase angle has been identified as a valid predictor of muscle mass changes during the competitive soccer season. Considerably, the phase angle represents a parameter that can be measured directly through bioelectrical impedance analysis, and it is independent of predictive equations such as those that quantify muscle mass.

**Abstract:**

The present study compared changes in body composition during the COVID-19-associated lockdown with the same period of the following season in elite soccer players. Fifteen elite male soccer players (30.5 ± 3.6 years.) underwent a bioelectrical impedance analysis (BIA) before (end of February) and after (end of May) the lockdown, which occurred during the 2019/2020 season, and at the same period during the following competitive season in 2020/2021, when restrictions were lifted. Fat and muscle mass were estimated using predictive equations, while phase angle (PhA) and bioelectrical impedance vector analysis (BIVA) patterns were directly measured. After lockdown, fat mass remained unchanged (*p* > 0.05), while muscle mass (95%CI = −1.12/−0.64; ES = −2.04) and PhA (95%CI = 0.51/−0.24, ES = −1.56) decreased. A rightward displacement of the BIVA vector was also found (*p* < 0.001, ES = 1.50). After the same period during the regular season, FM% and muscle mass did not change (*p* > 0.05), while the PhA increased (95%CI = 0.01/0.22; ES = 0.63). A leftward vector displacement (*p* < 0.001, ES = 1.05) was also observed. The changes in muscle mass correlated with changes in PhA (“lockdown” season 2019/2020: ß = −1.128, *p* = 0.011; “regular” season 2020/21: ß = 1.963, *p* = 0.011). In conclusion, coaches and strength conditioners should monitor muscle mass in soccer players during detraining periods as this parameter appears to be mainly affected by changes in training plans.

## 1. Introduction

The coronavirus disease 2019 (COVID-19) pandemic has had far-reaching social and health implications affecting the worldwide population, including athletes [1]. Soccer players experienced an initial lockdown, during which traditional training activities were suspended and players had to train individually at home. During this lockdown, drastic change in training plans led to a reduction in training activities, mimicking a detraining condition [2], consequently resulting in a reduction in performance parameters [3,4]. 

Previous studies with professional soccer players have shown that the detraining that occurs during the off-season break may impair body composition, increasing fat mass (FM) and reducing fat-free mass [5,6]. As for detraining context, the lockdown was also shown to affect body composition, increasing FM [4]. However, no further information on the effects of lockdown on body composition is available in soccer players. This may be of interest, since changes in fat-free mass, which includes muscle mass and body fluids, could negatively affect tolerance to high training exposure, possibly increasing the risk of injury [7]. In addition, muscle mass seems related to anaerobic performance in soccer players, given that its decrements correlated to decrements in sprinting and jumping capacity [8]. Therefore, monitoring these components of fat-free mass may also be relevant. 

Among the methods for assessing body composition, bioelectrical impedance analysis (BIA) has recently gained relevance, especially in soccer [9,10,11,12,13,14,15], where field and user-friendly methods are warranted [16]. Through the measurement and use of bioelectrical resistance (R) and reactance (Xc) in predictive equations, quantification of a wide range of body composition parameters is possible [17,18]. 

The combined evaluation of R and Xc as a vector within a graph results in a qualitative evaluation of body composition, defined as bioelectrical impedance vector analysis (BIVA) [19]. In BIVA, the position of the vector is interpreted in relation to its lateral and vertical displacements, which are reflected by phase angle (PhA) and vector length, respectively [15,20,21,22]. Several studies on soccer players have shown that PhA is related to sprint performance [11,12] and that BIVA patterns allow for the evaluation of nutritional status and physical condition during the competitive season [13,21,23,24]. 

Considering that a decline in body composition features impairs health and sports performance, exploring the effect of the COVID-19 lockdown could help practitioners better understand which parameters of body composition are most affected during a detraining period in soccer. Therefore, the present study aimed to examine whether or not BIA-derived FM and muscle mass and PhA were affected during the pandemic-associated lockdown in Series A soccer players. We compared the lockdown period with the same in-season period during the following 2020/21 regular season. We hypothesized that the lockdown affected body composition parameters as detected by BIA and BIVA. 

## 2. Materials and Methods

### 2.1. Participants

A total of 15 male soccer players (age 30.5 ± 3.6 years.; body mass 79.6 ± 7.6 kg; height 1.82 ± 0.1 m), currently competing in the Italian First division team (Serie A), voluntarily participated in the study. The exclusion criteria were age <18 years and muscle injury in the previous 6 months. After a detailed explanation of the procedures, participants signed informed consent. All research procedures were reviewed and approved by the Bioethics Committee of the University of Milan (approval number: 32/16) and conformed to the Declaration of Helsinki (1964 and further updates) concerning studies involving human subjects. 

### 2.2. Study Design and Procedures

The present investigation was designed as an observational, two-condition, two-time, one-group study. The exclusion criteria were age <18 years and muscle injury in the previous 6 months. The initial assessments were performed at the end of February 2020 as scheduled during the regular season, immediately before the COVID-19 lockdown. The post-lockdown assessments were performed at the end of May 2020, when the Italian Government lifted the restrictions. During the following 2020/21 season, we repeated the same assessment in the same period, i.e., end of February 2021 and end of May 2021. After the 2020 season, a total of 15 participants underwent all assessments and were included in analysis. 

Table 1 provides an overview of training contents within a typical weekly routine during both lockdown and competitive periods. During lockdown, each player followed an individualized nutritional and supplementation plan developed by the team’s nutritionist and adjusted every week. 

All anthropometric and BIA measurements were made in a resting and fasted state at least 24 h after the last exercise session and were profiled by an accredited and trained anthropometrist (T.B.). Height was recorded to the nearest 0.1 cm with a standing stadiometer (Seca 217, Basel, Switzerland), and body mass was measured to the nearest 0.1 kg with a high-precision mechanical scale (Seca 877, Basel, Switzerland). Body mass index (BMI) was calculated as the ratio of body mass to height squared (kg/m^2^). The impedance measurements were performed with a previously validated [19,20] bioimpedance analyzer (BIA 101 Anniversary, Akern, Florence, Italy) at a frequency of 50 kHz. Before each testing session, the analyzer was checked with a calibration circuit of known impedance (resistance = 500.0 Ω; reactance = 0.1 Ω; 0.9% error). The participants were assessed in the supine position with legs (45° compared to the median line of the body) and arms (30° from the trunk) abducted. After cleansing the skin with alcohol, two electrodes were placed on the right hand and two on the right foot. Bioimpedance values were analyzed according to the BIVA methods [16,19] and analyzed in relation to the distribution of the reference population (tolerance ellipses of Serie A soccer players) [9]. PhA was calculated as the arctangent of Xc/R*180°/π. Quantitative body composition was evaluated according to a three-compartment tissue model where body mass is given by the sum of FM, muscle mass, and residual components [16]. Body composition parameters were estimated using bioimpedance-derived equations [18,25] as follows:

%FM = [(Body mass − fat-free mass)/body mass] × 100, with fat-free mass = −2.261 + 0.327 × H^2^/R + 0.525 × body mass + 5.462 × 1

Muscle mass (kg) = −4.211 + (0.267 × H^2^/R) + (0.095 × body mass) + (1.909 × s1) + (−0.012 × age) + (0.058 × Xc)

### 2.3. Statistical Analysis

Data were analyzed using SPSS v. 27.0 (SPSS, IBM Corp., Armonk, NY, USA). The Kolmogorov–Smirnov test was used to ensure normal distribution of data. A one-way analysis of variance (ANOVA) was used to assess whether participants differed in the investigated parameters at baseline. A condition x time repeated-measures ANOVA was performed to determine changes in dependent parameters over time (two levels: PRE and POST) and condition (two levels: lockdown and regular seasons). Multiple comparisons were performed using Bonferroni’s correction. The paired, one-sample Hotelling’s T^2^ test, a multivariate extension of the Student’s *t*-test for paired data, was performed to determine if the changes in BIVA vectors were different from zero (null vector). Partial eta squared (*η*_p_^2^) was calculated to estimate the degree of variance of the dependent factor due to independent factors and interpreted as follows: <0.059: small; 0.06 to 0.12: medium; >0.13: large [26]. Cohen’s *d* effect size (ES) with 95% confidence interval (CI) was reported for significant pairwise comparisons, and Mahalanobis distance (D^2^), which represents a multivariate measure of effect and a multivariate measure of distance, was calculated to determine the magnitude of changes in the mean group vectors. Cohen’s ES was interpreted according to the following Hopkins’ recommendations: 0–0.19: trivial; 0.20–0.59: small; 0.60–1.19: moderate; 1.20–1.99: large; ≥2.00: very large [27]. Mahalanobis D^2^ was interpreted according to the following Stevens’s [28] guidelines: 0.25–0.49: small; 0.5–0.99; ≥1: large. Single and multiple regression analyses were performed to determine the correlation between the changes in FM% and muscle mass, considering the age of participants as an independent variable in the multiple regression. Data were reported as mean ± standard deviation, and significance was set at *p* < 0.05.

## 3. Results

No between-group difference (*p* > 0.05) in body mass or BMI was found at baseline. Table 2 shows change over time for each condition and statistical analysis for all dependent parameters. A time effect (*p* < 0.05) was found for body mass and BMI that decreased from PRE to POST during the “lockdown” season 2019/2020 (Table 2).

### 3.1. Quantitative Analysis

No between-group difference (*p* > 0.05) in FM% and muscle mass was found at baseline. Table 2 shows change over time for each condition and statistical analysis for all dependent parameters. A time effect (*p* < 0.01) was observed for muscle mass that decreased from PRE to POST during the “lockdown” season 2019/2020 (Table 2).

### 3.2. Qualitative Analysis

**Figure 1:**
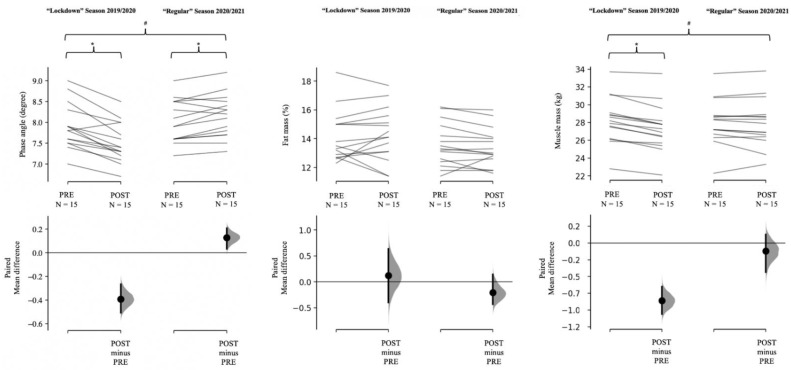
Paired mean differences for the comparisons are shown in the above Cumming estimation plots. Raw data are plotted on the upper axes; each paired set of observations is connected by a line. On the lower axes, each paired mean difference is plotted as a bootstrap sampling distribution. Mean differences are depicted as dots; 95% confidence intervals are indicated by the ends of the vertical error bars. * = significant (*p* < 0.05) time effect; # = significant (*p* < 0.05) condition by time interaction.

No between-group difference (*p* > 0.05) in R/H, Xc/H, or PhA was found at baseline. Table 2 shows change over time for each condition and statistical analysis for all dependent parameters. A time effect (*p* < 0.05) was observed for R/H, Xc/H, PhA, and muscle mass (Table 2). Figure 1 shows individual data for PhA, FM%, and muscle mass during the “lockdown” and the “regular” seasons.

**Figure 2:**
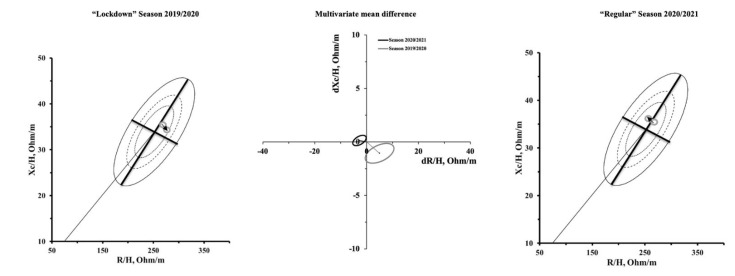
R-Xc and paired graphs for multivariate changes in specific resistance and reactance are shown on the left and right sides. In the R-Xc graphs, bioimpedance data are plotted on the tolerance ellipses of the reference population [9]. In the middle of the figure, mean vector displacements with 95% confidence ellipses are shown.

Multivariate analysis of the combined change in R/H and Xc/H showed differences from PRE to POST in both seasons, as shown in Figure 2. During the “lockdown” season 2019/2020, there was a rightward displacement of the BIVA vector from PRE to POST (T^2^ = 33.7, F = 15.7, *p* < 0.001, D^2^ = 1.50), due to a simultaneous reduction in R/H and Xc/H (Figure 2). A leftward BIVA vector displacement was observed during the “regular” season 2020/2021 from PRE to POST (T^2^ = 16.6, F = 7.7, *p* < 0.001, D^2^ = 1.05) due to an increase in Xc/H and a decrease in R/H (Figure 2).

### 3.3. Correlations between Qualitative and Quantitative Data

**Figure 3:**
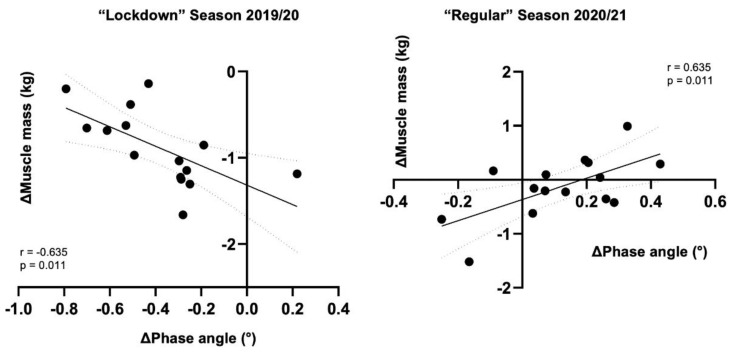
Scatterplots showing the associations between change in phase angle and change in muscle mass.

During both seasons, changes in PhA were correlated with changes in muscle mass (“lockdown” season 2019/2020: R^2^ = 0.403, ß = −1.128, *p* = 0.011; “regular” season 2020/21: R^2^ = 0.404, ß = 1.963, *p* = 0.011) (Figure 3). PhA remains a significant predictor even when adjusted for age (“lockdown” season 2019/2020: R^2^ = 0.404, ß = −1.134, *p* = 0.019; “regular” season 2020/21: R^2^ = 0.404, ß = 1.964, *p* = 0.015). No significant (*p* > 0.05) correlation was found for PhA and FM%.

## 4. Discussion

The aim of the present study was to examine whether or not bioelectrical FM%, muscle mass, and PhA were affected during the COVID-19 lockdown in Serie A soccer players. The main findings indicated that (i) FM% was not affected during home confinement but decreases in muscle mass were observed during the quarantine period; (ii) a decrease in PhA was found after the quarantine period, while an increase occurred when the same participants were tested during the successive regular season. Additionally, the change in PhA was correlated with change in muscle mass in both “lockdown” 2019/2020 and “regular” 2020/2021 seasons. To the best of our knowledge, this is the first study that examined the effect of the COVID-19 lockdown on a wide range of body composition parameters in a group of elite soccer players. 

The COVID-19 lockdown did not lead to any change in FM%, as also happened for the same period during the regular season. The exceptionality of the pandemic context and its impact on sports has been extensively studied recently, albeit only one study assessed body composition in soccer players [4]. Contrarily to what was observed here, the authors observed an increase in FM% after the lockdown [4]. However, first, FM% was estimated using skinfolds, so different procedures may somehow explain this inconsistency [29].

Second, the authors included lower-level soccer players, so it is possible that the nutritional and training strategies during lockdown were less controlled by the team staff. While FM% remained unchanged, lockdown led to a very large reduction in muscle mass. Such a reduction was probably the result of inadequate training stimulus sustained during forced home confinement. In accordance, previous studies have shown a reduction in fat-free mass in soccer players following the off-season period [5,30]. It should be noted that fat-free mass is not equivalent to muscle mass, but includes a wide range of elements (e.g., bone) not related to any physical capacity [16]. Having assessed muscle mass here allows the determination of a body composition parameter strictly related to sports performance in soccer players [31,32]. Indeed, increasing and preserving muscle mass is warranted across seasons [33], and the data collected here during the successive regular in-season period seems to confirm that. In this regard, the detraining-like lockdown period was already associated with deleterious effects on a wide range of neuromuscular and performance parameters in soccer players [3,34,35]. As such, the lockdown brought unfavorable changes in body composition in soccer players. Interestingly, muscle mass returned to baseline values at the end of the 2019/2020 season, showing how resumption of training and change in lifestyle can be decisive for the maintenance of muscle mass.

The PhA is calculated as the arctangent between R and Xc and is graphically reflected as the distance of the vector from the X-axis in BIVA [16]. Its interpretation allows qualitative estimation of the intracellular/extracellular water ratio, a biomarker of cellular integrity [20]. In the present study, the PhA decreased during the “lockdown” period, represented graphically by a rightward vector displacement, while it did not change during the successive regular season. In this regard, it has previously been shown that PhA remained stable during the in-season period [13]. The decrease in PhA can be ascribed to compromised cellular health, an accumulation of extracellular fluids, or a loss of body cell mass [13,24,36,37]. This may, in turn, depend on the loss of muscle mass observed during the detraining-like period. Indeed, we found a correlation between the simultaneous decrease in PhA and muscle mass during the lockdown. In contrast, previous studies have shown increases in PhA following the pre-season period in soccer players [9,13], as well as in other sports [21,38], possibly representing an increase in muscle mass, although the authors did not calculate the correlation. This evidence can be useful to practitioners who try to interpret changes in PhA and, therefore, in vector position in BIVA during the competitive period. As such, BIVA may be assessed regularly to evaluate body composition in sports practice. In this regard, phase angle and BIVA patterns represent direct bioelectrical measures, and this allows for the avoidance of predictive equations such as those used for quantifying fat and muscle mass.

The present study has some limitations. First, we acknowledge that the sample size is limited; however, it refers to elite soccer players, who are not easily recruited in practice. Second, the participants may have trained differently during home confinement, as the coach provided them with individualized training. Furthermore, different home confinement restrictions imposed by other countries may have resulted in different outcomes. Lastly, BIA is not identified as the gold standard for measuring fat and muscle mass. 

## 5. Conclusions

During the lockdown period experienced in the 2019/2020 season, elite soccer players showed no change in FM%, while muscle mass decreased along with PhA. When returning to regular training in the same period of the successive season, muscle mass remained stable, while PhA increased. The change in bioelectric properties resulted in a rightward shift of the BIVA vector during the lockdown, and a leftward displacement during the regular season. In soccer practice, BIA and BIVA could be regularly used to assess body composition during the season. In particular, BIVA represents a qualitative analysis of body composition, which, through bioelectrical vector and PhA assessment, allows the evaluation of directly obtained measurements. The results of this study could lead coaches and strength conditioners to monitor muscle mass during detraining periods, since this parameter appears to be mainly affected in elite soccer players.

## Figures and Tables

**Table 1:** Overview of training contents during the COVID-19 lockdown and competitive period.

Period	No of Training Weeks	No of Training Sessions/Matches	Weekly Training Contents *
Competitive	14	84	*Monday:* rest day. *Tuesday and Thursday:* sessions based on TT and aerobic training. *Wednesday:* session based on a combination of strength-related stimuli with a special emphasis on lower limbs. *Friday and Saturday:* sessions based on a combination of low-intensity TT, attacking and defending maneuvers, and SAQ training. *Sunday:* match day.
Lockdown	14	84	*Monday, Wednesday, and Saturday:* sessions based on a combination of aerobic drills suitable for the home-confinement condition. *Tuesday and Thursday:* sessions based on a combination of aerobic (mainly running/cycling-based MIIT) training and strength-related stimuli suitable for the home-confinement condition. *Friday:* sessions based on a combination of aerobic drills (mainly running/cycling-based LIT) suitable for the home-confinement condition. *Sunday:* rest day.

Note: TT = technical and tactical, HIIT = high-intensity interval training, MIIT = moderate-intensity interval training, LIT = low-intensity interval training, SAQ = speed, agility, and quickness, and COD = change of direction. * Additional individual training, injury prevention program, and warm-up sessions are not included.

**Table 2:** Baseline (PRE) and post values (mean ± standard deviation) of dependent parameters are shown. Differences over time are reported as mean with 95% confidence interval (CI). Effect size is also reported.

Variable		“Lockdown” 2019/2020 Season	“Regular” 2020/2021 Season	Time Effect	Condition x Time Interaction
Body mass (kg)	PRE	79.6 ± 7.6	79.7 ± 7.9	F = 4.40, *p* = 0.045, *η*_p_^2^ = 0.136	F = 8.26, *p* = 0.008, η_p_^2^ = 0.228
	POST	78.6 ± 7.8 *	79.8 ± 7.9		
	95% CI	−1.66/−0.22	−0.21/0.51		
	Effect size	−0.72	0.22		
Body mass index (kg/m^2^)	PRE	23.7 ± 1.0	23.7 ± 1.1	F = 1.43, *p* = 0.242, *η*_p_^2^ = 0.049	F = 9.66, *p* = 0.004, *η*_p_^2^ = 0.257
	POST	23.4 ± 1.1 *	23.8 ± 1.1		
	95% CI	−0.06/−0.49	−0.05/0.30		
	Effect size	−0.73	0.38		
R/H (Ohm/m)	PRE	258.9 ± 22.4	253.7 ± 19.7	F = 1.09, *p* = 0.305, *η*_p_^2^ = 0.038	F = 13.11, *p* = 0.001, *η*_p_^2^ = 0.319
	POST	263.9 ± 23.5 *	250.9 ± 19.3 *		
	95% CI	0.83/9.15	−4.69/−0.81		
	Effect size	0.66	−0.78		
Xc/H (Ohm/m)	PRE	35.6 ± 3.6	35.4 ± 3.1	F = 6.53, *p* = 0.016, *η*_p_^2^ = 0.189	F = 10.88, *p* = 0.003, *η*_p_^2^ = 0.028
	POST	34.6 ± 3.5 *	35.6 ± 3.1		
	95% CI	−1.75/−0.37	−0.22/0.49		
	Effect size	−0.85	0.20		
PhA (degree)	PRE	8.0 ± 0.5	7.9 ± 0.5	F = 10.74, *p* = 0.003, *η*_p_^2^ = 0.277	F = 39.24, *p* < 0.001, *η*_p_^2^ = 0.584
	POST	7.5 ± 0.5 *	8.1 ± 0.5 *		
	95% CI	−0.51/−0.24	0.01/0.22		
	Effect size	−1.56	0.63		
Fat mass (%)	PRE	14.1 ± 1.7	13.6 ± 1.5	F = 0.51, *p* = 0.823, *η*_p_^2^ = 0.002	F = 1.31, *p* = 0.261, *η*_p_^2^ = 0.045
	POST	14.3 ± 1.9	13.4 ± 1.3		
	95% CI	0.69/−0.41	−0.51/0.10		
	Effect size	0.13	−0.35		
Muscle mass (kg)	PRE	28.2 ± 2.3	28.1 ± 2.3	F = 29.54, *p* < 0.001, *η*_p_^2^ = 0.513	F = 16.29, *p* < 0.001, *η*_p_^2^ = 0.368
	POST	27.4 ± 2.7 *	27.9 ± 2.7		
	95% CI	−1.12/−0.64	−0.45/0.19		
	Effect size	−2.04	−0.22		

Note: * = *p* < 0.05 vs. PRE. R/H: resistance adjusted for stature, Xc/H: reactance adjusted for stature, PhA: phase angle.

## Data Availability

The data presented in this study are available on request from the corresponding author. The data are not publicly available due to privacy.
